# Viral load testing among pregnant women living with HIV in Mutare district of Manicaland province, Zimbabwe

**DOI:** 10.1186/s12981-022-00480-1

**Published:** 2022-11-16

**Authors:** Christine Chiedza Chakanyuka Musanhu, Kudakwashe C. Takarinda, Jawaya Shea, Inam Chitsike, Brian Eley

**Affiliations:** 1 World Health Organization Country Office, Highlands, P.O.Box HG 430, Harare, Zimbabwe; 2 AIDS & TB Department, Ministry of Health and Child Care, Harare, Zimbabwe; 3 Department of Paediatrics and Child Health, University of Cape Town, Cape Town, South Africa; 4 College of Health Sciences, University of Zimbabwe, Harare, Zimbabwe; 5 Paediatric Infectious Diseases Unit, Red Cross War Memorial Children’s Hospital, Cape Town, South Africa

**Keywords:** Pregnancy, HIV PMTCT, HIV viral load monitoring, HIV/AIDS, Human Immunodeficiency Virus

## Abstract

**Background:**

Viral load (VL) monitoring of pregnant women living with HIV (PWLHIV) and antiretroviral therapy (ART) may contribute to lowering the risk of vertical transmission of HIV. The aims of this study were to assess the uptake of HIV VL testing among PWLHIV at entry to the prevention-of-mother-to-child transmission (PMTCT) services and identify facilitatory factors and barriers to HIV VL access.

**Methods:**

A retrospective, cross-sectional study was conducted at 15 health facilities in Mutare district, Manicaland Province, Zimbabwe from January to December 2018. This analysis was complemented by prospective interviews with PWLHIV and health care providers between October 2019 and March 2020. Quantitative data were analysed using descriptive and inferential statistical methods. Risk factors were evaluated using multivariate logistic regression. Open-ended questions were analysed and recurring and shared experiences and perceptions of PWLHIV and health care providers identified.

**Results:**

Among 383 PWLHIV, enrolled in antenatal care (ANC) and receiving ART, only 121 (31.6%) had a VL sample collected and 106 (88%) received their results**.** Among these 106 women, 93 (87.7%) had a VL < 1000 copies/mL and 77 (73%) a VL < 50 copies/mL. The overall median duration from ANC booking to VL sample collection was 87 (IQR, 7–215) days. The median time interval for the return of VL results from date of sample collection was 14 days (IQR, 7–30). There was no significant difference when this variable was stratified by time of ART initiation. VL samples were significantly less likely to be collected at local authority compared to government facilities (aOR = 0.28; 95% CI 0.16–0.48). Barriers to VL testing included staff shortages, non-availability of consumables and sub-optimal sample transportation. Turnaround time was prolonged by the manual results feedback system.

**Conclusions and recommendation:**

The low rate of HIV VL testing among PWLHIV in Mutare district is a cause for concern. To reverse this situation, the Ministry of Health should consider interventions such as disseminating antiretroviral guidelines and policies electronically, conducting regular PMTCT mentorship for clinical staff members, and utilising point of care testing and telecommunication devices like mHealth to increase uptake of VL testing and improve results turnaround time.

**Supplementary Information:**

The online version contains supplementary material available at 10.1186/s12981-022-00480-1.

## Introduction

The human immunodeficiency virus (HIV) remains a leading cause of death among women during pregnancy and the postpartum period, especially in areas of high HIV prevalence [1]. Maternal plasma HIV viral load (VL) is the principal determinant of HIV vertical transmission [2–6]. Effective interventions during pregnancy, labour, delivery and breastfeeding reduces vertical transmission rates from 15 to 45% to below 5% [7].

There is no VL concentration above or below which vertical transmission of HIV always or never occurs [5, 8]. In published studies, the perinatal HIV transmission rate was significantly higher in pregnant women with VL concentrations of 50–400 copies/mL near delivery compared to concentrations < 50 copies/mL [9, 10]. Furthermore, among pregnant women living with HIV (PWLHIV) and receiving ART the risk of perinatal transmission of HIV is proportional to the maternal VL concentration [11]. Thus, while WHO uses a maternal VL of < 1000 copies/mL to define low risk of perinatal transmission of HIV, transmission of HIV may still occur below this VL concentration [12–14].

Since 2016 WHO guidelines have recommended that all PWLHIV should have an HIV VL test at 36 weeks gestational age to determine the risk status of infants and plan for their optimal management [7]. This intervention is meant to contribute to the reduction of vertical transmission and the elimination of mother to child transmission (eMTCT). Viral load monitoring during pregnancy can help guide the health care provider to institute more intensive adherence interventions if the pregnant woman is found to have a VL > 1000 copies/mL [3, 15]. Additionally, VL monitoring will aid the diagnosis and confirmation of ART failure [7]. Thus, VL monitoring plays an important role in the optimal management of prevention of mother to child transmission of HIV (PMTCT) interventions and is superior to immunological monitoring [16].

Zimbabwe, one of the high HIV burden countries in sub-Saharan Africa (SSA) with a prevalence of 14%, [17] adopted an amended version of the 2016 WHO guidelines [18]. Mutare district in Manicaland province started offering HIV VL testing in June 2015 with support from the Ministry of Health and Médecins Sans Frontières (MSF). By end of 2019, HIV VL services had been cascaded to 42 health facilities in Mutare district and included VL testing for pregnant and breastfeeding women. However, VL testing coverage in pregnant and breastfeeding women living with HIV has remained low at 63% at end of 2017 [19]. The aims of this study were to assess the uptake of HIV VL testing among PWLHIV at entry into the PMTCT services and identify facilitatory factors and barriers to HIV VL access at selected health facilities in Mutare district, Manicaland Province, Zimbabwe.

## Methods

### Study design

This cross-sectional study comprised two components, a retrospective descriptive analysis of demographic, clinical and VL data of PWLHIV who attended 15 health facilities in Mutare district between 1 January and 31 December 2018, sourced from ANC and ART registers, and patient booklets. Additionally, key informant interviews of PWLHIV at entry to the PMTCT services and health care providers were conducted prospectively at the same 15 health facilities in Mutare district between 1 October 2019 and 31 March 2020.

### Study setting

Manicaland province is the largest of 10 provinces and situated in eastern Zimbabwe. It has a population of 1,862,000 with 983,000 being females, of whom 836,000 are women of childbearing potential [17]. The province has nine administrative districts, three of these (Mutare, Chipinge and Makoni) have rural and urban populations. The HIV prevalence in adults aged 15–49 years in the province is 10.5% [20].

### Study sites

The 15 health facilities (study sites) were selected from 51 health facilities providing ANC services in Mutare district. The 51 health facilities were grouped by strata based on level of service provision and size i.e., clinic, rural/mission hospital, and tertiary hospital and geographical location, namely rural or urban location. The 15 study sites were then randomly selected by stratified sampling while ensuring representation by level of service provision and geographical location.

### Sample size estimation

For the retrospective component, the sample size was estimated using a formula for a prevalence study, assuming 50% of PWLHIV at the 15 research sites received a VL test during their ANC visits, a confidence interval of 95% and study precision of 5%. The calculated sample size was 384. At each of the 15 study sites all PWLHIV who received ANC during the enrolment period (1 January to 31 December 2018) were listed sequentially in an excel spreadsheet according to their ANC numbers. The target number of PWLHIV for each site (Additional file 1: Table S1) was randomly selected. At sites where the target sample was equal to the number of PWLHIV who received ANC during the study period, all were included.

The structured prospective interviews using standardised questionnaires were conducted to provide more in-depth information from beneficiaries of ANC services and health care providers. The PWLHIV who were prospectively interviewed were a different group from those included in the retrospective analysis. For the prospective interviews there was no sample size estimation. Instead, the plan was to interview a minimum of one nursing professional at each of the 15 study sites, at least one PWLHIV per study site, and one laboratory scientist at the provincial laboratory. These individuals were selected by convenience sampling. For each of the study sites the prospective interviews were conducted on the same day that the retrospective data were abstracted.

### Data collection

For the retrospective component, paper-based ANC and ART registers and patient booklets were reviewed at the 15 study sites and relevant data were extracted on paper-based datasheet and transferred to an electronic database in Epidata version 3.1 [21].

The responses of the prospectively interviewed PWLHIV and health care providers were manually entered in standardised questionnaire sheets and thereafter transferred to the electronic database in Epidata version 3.1 [21].

### Data analysis

Quantitative data were exported to Stata version 15 (StataCorp, College Station, Texas, USA) for data cleaning and analysis [22]. Data were summarised using numbers and percentages for categorical variables, and medians and interquartile ranges for non-normally distributed continuous variables. The Wilcoxon rank-sum test was used to compare medians of non-normally distributed continuous variables. The Chi-squared test and Fischer’s exact test were used to compare categorical variables. Univariate- and multivariate-adjusted odds ratios [(a)OR] and their 95% confidence intervals (CIs) for factors associated with VL collection were explored using logistic regression. The multivariate logistic regression model was built, using variables which on univariate analysis had a *p-*value < 0.25. Parity and type of health facility were excluded from the logistic regression model because of their collinearity with gravidity and type of health facility ownership, respectively. Levels of significance were set at 5%. Structured questionnaires included open-ended questions that explored perceptions and experiences of VL testing. Open-ended questions were analysed, and recurring or shared experiences and perceptions identified. An independent reviewer (JS) supported the researcher’s (CCCM) interpretation of these responses.

## Results

### Study participants

**Fig. 1:**
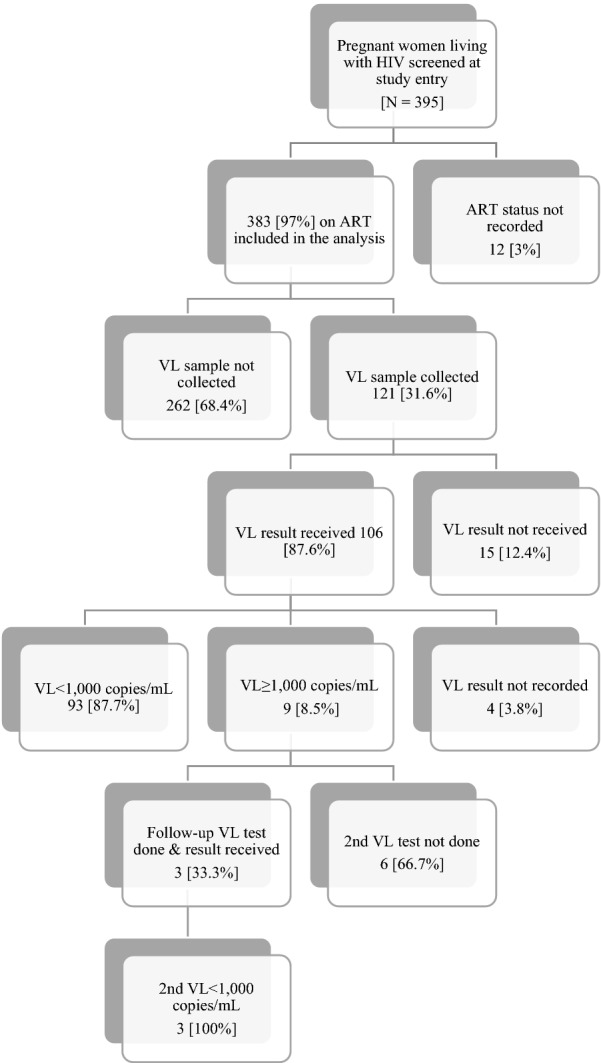
Study algorithm describing the participants, and the number and proportion who had viral load (VL) samples collected and who received their VL results

Data was obtained on 383 PWLHIV who were either on ART or ART naïve at the time of enrolment into ANC at the 15 study sites, representing 99.7% of the estimated sample size of 384 (Fig. 1 and Additional file 1: Table S1). Prospective interviews were completed by 12 nurses at 12 of the study sites, 1 laboratory scientist at the provincial.

### Characteristics of study participants

The characteristics of the 383 women enrolled in the retrospective component of this study are summarised in Table 1. Nearly half of these women (46.7%) were aged 30–49 years. Only 42 (11.0%) of the PWLHIV presented for ANC booking during the first trimester. Among all PWLHIV with recorded ART status, 256 (66.8%) were already receiving ART by the time they were booked for ANC whilst 127 (33.1%) were newly tested HIV positive and started on ART. Most women were enrolled at primary health care facilities (82.5%) and most at health facilities belonging to a local authority (68.1%).

**Table 1:** Characteristics of pregnant women living with HIV attending antenatal care in Mutare district in 2018

Characteristic	All pregnant women living with HIV on ART	Women who did not have a VL performed	Women who had VL performed	p value^a^
	N = 383	N = 262	N = 121	
	n (%)	n (%)	n (%)	
*Age category (in years)*				
10–14	3 (0.8)	1 (0.4)	2 (1.7)	0.125
15–19	24 (6.3)	21 (8.0)	3 (2.5)	
20–24	73 (19.1)	51 (19.5)	22 (18.2)	
25–29	86 (22.5)	55 (21.0)	31 (25.6)	
30–49	179 (46.7)	120 (45.8)	59 (48.8)	
Not recorded	18 (4.7)	14 (5.3)	4 (3.3)	
*Gravidity*				
1	63 (16.5)	46 (17.6)	17 (14.1)	0.109
2–3	190 (49.6)	134 (51.2)	56 (46.3)	
4–5	115 (30.0)	69 (26.3)	46 (38.0)	
6–7	13 (3.4)	11 (4.2)	2 (1.7)	
Not recorded	2 (0.5)	2 (0.8)	0 (0)	
*Parity*				
0	71 (18.5)	54 (20.6)	17 (14.1)	0.204
1–2	193 (50.4)	130 (49.6)	63 (52.1)	
3–4	105 (27.4)	66 (25.2)	39 (32.2)	
5–6	12 (3.1)	10 (3.8)	2 (1.7)	
Not recorded	2 (0.5)	2 (0.8)	0 (0)	
*Gestational age at ANC booking*				
0–12 weeks	42 (11.0)	12 (9.9)	30 (11.5)	0.137
13–26 weeks	235 (61.4)	83 (68.6)	152 (58.0)	
27 + weeks	83 (21.7)	19 (15.7)	64 (24.4)	
Not recorded	23 (6.0)	16 (6.1)	7 (5.8)	
*ART status at ANC booking*				
On ART prior to ANC booking	256 (66.8)	171 (65.3)	85 (70.3)	0.336
Started ART after ANC booking	127 (33.2)	91 (34.7)	36 (29.8)	
*Type of health facility*				
Primary health care clinic	316 (82.5)	225 (85.9)	91 (75.2)	0.008
Rural hospital	35 (9.1)	16 (6.1)	19 (15.7)	
District/mission hospital	32 (8.4)	21 (8.0)	11 (9.1)	
*Health facility ownership*				
Government	91 (23.8)	43 (16.4)	48 (39.7)	< 0.01
Local authority	261 (68.2)	199 (76.0)	62 (51.2)	
Faith-based (mission)	31 (8.1)	20 (7.6)	11 (9.1)	

*ART* Antiretroviral therapy, *VL* Viral load, *ANC* Antenatal care

^a^Comparison of women who had VL performed and those who did not have a VL performed

### Viral load testing

Among the 383 PWLHIV in ANC and receiving ART, VL blood samples were collected for 121 (31.6%), of whom 106 (87.6%) received their VL results (Fig. 1). Among these 106 women, 93 (87.7%) had VL results of < 1000 copies/mL–77 had VL results < 50 copies/mL, 4 had VL results between 50 and 199 copies/mL and 11 had VL results between 200 and 999 copies/mL. Of the 9 women (8.5%) with VL results > 1000 copies/mL, three (33%) had follow-up VL tests done after enhanced adherence counselling (EAC). The follow-up VL results of all 3 women were < 1000 copies/mL.

The median duration between ANC booking and VL sample collection was significantly longer among those newly started on ART. However, the median duration for the return of VL results was not significantly different between the two groups (Table 2).

**Table 2:** Comparison of the time between ANC booking and VL sample collection, and the time between VL sample collection and receipt of results in pregnant women living with HIV on ART and those not on ART at the time of ANC enrolment

Time duration (in days)	Total		ART status at enrolment into ANC				p value*
			Already on ART		Not on ART		
	n	Median (IQR)	n	Median (IQR)	N	Median (IQR)	
Time from ANC booking to VL sample collection	100	87 (7–215)	67	50 (0–162)	33	207 (99–299)	< 0.001
Time from VL sample collection to receipt of result	52	14 (7–30)	36	10 (7–30)	16	17 (11–45)	0.174

*ANC* Antenatal care, *HIV* Human immunodeficiency virus, *ART* Antiretroviral therapy, *IQR* Interquartile range, *VL* Viral load

### Factors associated with viral load sample collection

In the univariate analysis, receiving care at a rural hospital compared to a primary health care facility [OR = 2.94; 95% CI 1.45–5.96] was significantly associated with VL collection. Furthermore, VL samples were significantly less likely to be collected at local authority facilities compared to government facilities [OR = 0.28; 95% CI 0.17–0.46]. On multiple logistic regression the association of VL collection at local authority facilities compared to government facilities remained statistically significant [aOR = 0.28; 95% CI 0.16–0.48]. Gravidity, parity, gestational age, and ART status at ANC booking were not associated with VL sample collection (Table 3).

**Table 3:** Factors associated with the collection of viral load samples among pregnant women living with HIV enrolled into antenatal care in Mutare District, Zimbabwe

Characteristic	n	VL sample collected	OR (95% CI)	aOR (95% CI)
		n (%)		
Total	383	121 (31.6)	–	–
*Age (in years)*				
10–14	3	2 (66.7)	4.07 (0.36–45.77)	2.05 (0.16–26.79)
15–19	24	3 (12.5)	0.29 (0.08–1.01)	0.27 (0.07–1.11)
20–24	73	22 (30.1)	0.88 (0.49–1.58)	0.94 (0.46–1.92)
25–29	86	31 (36.1)	1.15 (0.67–1.97)	1.10 (0.61–1.99)
30–49	179	59 (33.0)	Reference	Reference
Not recorded	18	4 (22.2)	0.58 (0.18–1.84)	0.75 (0.22–2.55)
*Type of health facility*				
Primary health care clinic	316	91 (28.8)	Reference	Reference
Rural hospital	35	19 (54.3)	**2.94 (1.45–5.96)**	–
District/mission hospital	32	11 (34.4)	1.30 (0.60–2.79)	–
*Health facility management*				
Government	91	48 (52.8)	Reference	Reference
Local authority	261	62 (23.8)	**0.28 (0.17–0.46)**	**0.28 (0.16–0.48)**
Faith-based (mission)	31	11 (35.5)	0.49 (0.21–1.14)	0.52 (0.21–1.29)
*Gravidity*				
1	63	17 (27.0)	Reference	Reference
2–3	190	56 (29.5)	1.13 (0.60–2.14)	0.76 (0.36–1.62)
4–5	115	46 (40.0)	1.80 (0.92–3.52)	1.07 (0.44–2.57)
6–7	13	2 (15.4)	0.49 (0.10–2.45)	0.28 (0.05–1.63)
Not recorded	2	0 (0)	–	–
*Parity*				
0	71	17 (23.9)	Reference	Reference
1–2	193	63 (32.6)	1.54 (0.83–2.87)	–
3–4	105	39 (37.1)	1.88 (0.96–3.68)	–
5–6	12	2 (16.7)	0.64 (0.13–3.19)	–
Not recorded	2	0 (0)	–	–
*Gestational age at ANC booking*				
0–12 weeks	42	12 (28.6)	Reference	Reference
13–26 weeks	235	83 (35.3)	1.37 (0.66–2.81)	1.30 (0.59–2.86)
27 + weeks	83	19 (22.9)	0.74 (0.32–1.72)	0.68 (0.27–1.74)
Not recorded	23	7 (30.4)	1.09 (0.36- 3.33)	1.31 (0.40–4.26)
*ART status at ANC booking*				
On ART prior to ANC booking	256	85 (33.2)	Reference	Reference
Started ART after ANC booking	127	36 (28.4)	0.80 (0.50–1.27)	1.12 (0.32–1.84)

Bold values represent values that are statistically significant

*VL* Viral load, *OR* Odds ratio, *aOR* Adjusted odds ratio, *95% CI* 95% confidence interval, *ART* Antiretroviral therapy, *ANC* Antenatal care

### Structured interviews with pregnant women living with HIV

All 19 PWLHIV who participated in the prospective interviews reported that they had received information on VL testing during group health education talks provided before receiving ANC services, or during one-on-one consultation with a nurse or both. Twelve of the 19 (63.1%) women had a VL test done. Ten of 16 (62.5%) women who were interviewed understood what VL testing meant as substantiated by the following specific comments: Participant 10, a 42 year old woman diagnosed with HIV infection prior to her current pregnancy and already on ART stated that: “a viral load test detects the activity of the virus in one’s body”, while participant 18, a 29 year old woman with secondary education, presenting with her second pregnancy, newly diagnosed with HIV infection and started on ART stated that “a viral load test is taken to check if the ARVs I am taking are working and to check if I am responding to them”. However, four of the 16 (25%) women indicated that blood samples were taken but VL was not explained to them. The turnaround time (TAT) for VL results for ten women who responded ranged from 1 to 4 days. Thirteen of the 19 (68.4%) women understood the meaning of a low VL result; participant 14, a 29 year old woman having her second pregnancy stated that “a low VL result means that I am adhering to my treatment”; participant 11, a 32 year old woman pregnant for the third time, previously known to have HIV infection and on ART prior to her current pregnancy stated that “it means that the virus is being suppressed”; and participant 19, a 35 year old woman having her first pregnancy stated that “the virus strength is low, and my health is good”. Twelve of the 19 [63.1%] women understood the meaning of a high VL result.

Pregnant women living with HIV mentioned the following as factors that facilitated access to VL testing: VL sample collection synchronised with either ANC visits or antiretroviral (ARV) resupply days, free VL testing services, easy accessibility to the health facility and outreach services by nurses brought VL sample collection to their doorstep. The following barriers to accessing VL testing were cited by some PWLHIV: few nurses at the facility compared to the number of patients they serve meant that sometimes they deferred taking samples for VL testing, sometimes there were no supplies such as dry blood spot (DBS) consumables to enable the nurses to take the blood samples, some facilities were too far, hence PWLHIV could not access services on time, and some women could not access VL testing for religious reasons.

### Interviews with health care providers

Of the 12 health care providers interviewed, 6 (50%) were primary care nurses (PCNs), 4 (33.3%) registered general nurses (RGNs) and 2 (16.7%) were midwives by training. Four of 12 health facilities (33.3%), all local authority health facilities, charged user fees for ANC services of one hundred Zimbabwean Dollars (equivalent to 5 United States Dollars) per visit. Laboratory services were free in all 12 facilities.

Ten of 12 (83.3%) health care providers were aware of the existence of the 2016 Zimbabwean ARV guidelines. Seven of the 12 health facilities had two or more nurses trained on the new guidelines. Training information was not available for the five remaining clinics. Ten (83,3%) nurses indicated that the guidelines were easy to understand. Although all twelve health care providers had received hard copies of the 2016 ARV guidelines it took up to 2 years for the guidelines to reach these facilities. Nine of 12 (75%) of the nurses were knowledgeable about the ARV guidelines, including the VL testing requirements. Eight of the 12 (66.7%) nurses provided feedback to their patients on their VL results during group education talks and the remaining four (33.3%) provided feedback during one-on-one consultations. Nine out of twelve (75%) facilities used dry blood samples (DBS). Only paper-based results were distributed to all 12 facilities. None of these facilities received their results via telephone or text messages. Viral load TAT was 1 to 2 weeks for six of the health facilities and between 2 and 8 weeks for the remaining six facilities.

### Interview with the laboratory scientist

Viral load testing is centralised at the provincial laboratory staffed by 10 laboratory scientists and one laboratory technologist, all of whom were trained on VL testing. At the time of the interview, 225 facilities in the province submitted VL samples to the provincial laboratory of which 42 (18.7%) were in Mutare District. On average, 11,500 VL samples were received by the laboratory every month, of which 1% were from PWLHIV. According to the laboratory, TAT for results was on average 1–2 weeks from receipt of sample to release of results. The sample rejection rate was around 1%. Laboratory challenges in the provision of VL testing services included shortage of laboratory staff particularly data clerks, sample sorters and laboratory scientists, limited operating space, and delays in submitting whole blood specimens from some facilities. The laboratory information management system was not linked to the clinical services health information system at the facilities. Consequently, results were not available at health facility level in real time.

### Health facility and patient related challenges

Health facility and patient related challenges faced from a health worker perspective in the provision of VL services are listed in Table 4.

**Table 4:** Health facility and patient related challenges encountered in the provision of viral load testing services

Health facility related problems
*Transport related reasons*
Lack of safe and reliable mode of transport for laboratory specimens Fuel shortages impeded sample collection Irregular sample collection from facilities Transport challenges adversely affected VL turnaround time
*Sample related issues*
Shortage of EDTA tubes for collecting whole blood samples for viral load testing High rejection rate as a result poor sample quality due to delays in transporting whole blood samples from facilities to the laboratory Not enough space for privacy to attend to a patient's needs (e.g., counselling a patient on HIV-related issues including need for viral load testing)
*Data issues*
Non-availability of viral load registers to document when viral load tests were conducted, when viral load results were received and if clients received their results. Nurses improvised and utilised notebooks which resulted in incomplete entries
For those facilities with electronic systems, systems were not customised to capture viral load results; hence pregnant women living with HIV who were in the ePMS would not have any results highlighted in the systems, even though results may have been available at the facility. The information system showed the patients as not having viral load tests done
**Patient-related problems**
Patients sometimes gave health care providers incorrect contact information
Challenges with contacting and follow-up of patients living outside the health facility catchment area
Some patients do not return for antenatal care visits and do not collect viral load results on time
Late booking for antenatal care and hence delays in having first VL tests
Failure of patients to understand their results

*EDTA* Ethylenediamine tetra acetic acid, *ePMS* electronic patient monitoring system

## Discussion

Antenatal care is the key health care entry point for pregnant women to access an array of health services for improving foetal and maternal outcomes [7]. WHO recommends that pregnant women have their first ANC contact within the first 12 weeks of gestation. In this study only 42 (11.0%) of PWLHIV presented for their ANC booking visits during the first trimester, lower than the findings of the Zimbabwe Demographic Health Survey (ZDHS, 2015/2016) which showed that 39% of PWLHIV and uninfected women booked in the first trimester. Another study completed in rural Mashonaland East province in 2017 showed that 29.2% of pregnant women booked during the first trimester, which is more than two-fold higher than this study, but still much lower than the national figure of 39% [23]. Late booking of PWLHIV implies that their HIV diagnosis and ART initiation could be delayed, increasing the risk of mother-to-child-transmission. A South African study observed that late ANC booking, and late ART initiation were associated with failure to achieve viral suppression in PWLHIV despite the services being free of charge. In this study delay in antenatal attendance was prevalent despite ANC and laboratory services being offered free of charge. Several studies in countries in the African region including South Africa [24, 25], Ethiopia [26] and Malawi [27] identified a spectrum of patient and health-related factors associated with late booking.

Among the PWLHIV enrolled into ANC in this study, only 31.6% had a VL test done. Although this coverage is comparable to studies done in the East and Southern African region, this is unacceptably low given that Manicaland province was among the first provinces in Zimbabwe to be supported to provide VL testing. Viral load testing in Manicaland province at the commencement of this service was initially focussed on pregnant and breastfeeding women, children and general clients with treatment failure then expanding to routine VL testing [28–31]. Viral load coverage at national level was higher at 44% and 54% in 2018 and 2019 respectively [32]. Another study in Zimbabwe recorded a VL testing coverage of 31.9% in a cohort of pregnant and breastfeeding women [33]. Viral load coverage was 63.1%, almost two-fold higher in 16 women interviewed for this study between October 2019 and March 2020. Although this coverage was higher than the 31.6% recorded in the retrospective component completed in 2018 the sample size was very small. Thus, further study of a larger, more representative sample is needed to determine whether VL testing coverage in pregnant women on ART has increased over time.

Women who were already on ART at the time of ANC booking had their VL tests done much earlier than the women who were newly started on ART. This was aligned to the Zimbabwean guidelines which stated that women who are already on ART would have a VL test done at first ANC booking, while for newly initiated women, VL testing was to be conducted 3 months after the first ANC booking. Of concern was that the median time from booking to having a VL test done for newly initiated women was 207 days meaning that these women had their VL test done a median of 6.9 months after ART initiation instead of the recommended 3 months after ART initiation.

The median TAT between VL specimen collection and receipt of VL results by the PWLHIV for this study was 14 days (IQR, 7–30); this is still unacceptably high though it compared well to the maximum TAT target for the programme of 14 days [34]. Point of care testing could shorten the TAT by providing same day results, thereby benefiting VL unsuppressed women as they can get their results during the same clinic visit and be managed appropriately. A study in Botswana concluded that use of point of care (POC) testing is a feasible and reliable option for VL monitoring, even in rural settings and that this would provide same-day VL results, allow for immediate assessment of virologic failure and reduce loss to follow-up [35].

Nicholas et al. [36] evaluated the outcomes of the first 4 years of routine VL monitoring using POC in rural clinics in Malawi and concluded that high VL coverage can be achieved followed by same-day test results and shorter time-to-switch to new ARV regimens. The game-changing potential of POC-based VL testing compared to conventional testing was also demonstrated [37, 38]. Our study showed that the major challenges were unreliable transport for specimens to the laboratory and long TAT. These challenges can be solved with the introduction and scale up of POC technology [39].

Of the PWLHIV who had VL testing, 87.6% received their results and of these 87.7% had a VL < 1000 copies/mL. This is a good outcome and indicates good adherence to ARVs among pregnant women living with HIV which can lead to reduction in MTCT. In our study of 106 who had VL testing 77 (72.6%) were virally suppressed to < 50 copies/mL. Similar high VL suppression rates (< 50 copies/mL) were documented in studies in Rwanda [40], Ethiopia [41, 42] and Nigeria [43].

Our results showed that VL specimens were less likely to be collected at local authority facilities compared to government facilities. This observation requires more research to establish the actual reasons for the difference in testing practices. A similar study by Atuhaire et al*.* [44] in 2015 among PWLHIV at five local authority health facilities also noted a difference in practices in VL testing among the facilities, however it was noted that more research was required to establish the cause of such differences. Lessons learned from the Therapeutic Solidarity and Initiatives against AIDS, OPP-ERA project in five West African countries showed that disparities in VL sample collection can be due to multiple determinants including the number, training and availability of health staff, size of the HIV cohort at the facility, and organisation of the sample collection circuit [45]. In that study enabling factors for VL sample collection included the provision of a one stop shop for HIV services or the supermarket approach, where the pregnant women receive all their services under one roof from the same health worker and on the same day, and the provision of free ANC services. Ease of collection and preparation of DBS versus whole blood sample collection also facilitated the collection VL samples. Age, gravidity, parity, and gestational age at ANC booking were not predictors for VL sample collection. The barriers affecting VL collection were mostly systemic and included staff shortages, non-availability of consumables and laboratory forms and weaknesses in sample transportation. Lecher et al*.* [46] noted similar challenges during monitoring of progress with scale-up of HIV VL in seven sub-Saharan African countries.

Findings from the interviews of nursing personnel indicated that they were aware of the national guidelines on VL testing in pregnant women. Despite possessing this knowledge, VL testing coverage was low. It was noted in a retrospective cohort analysis of patients eligible for routine VL testing between 2013 and 2017 in Malawi and a retrospective data review of routine reports from MSF-supported health facilities in Zimbabwe that despite health care providers knowledge of the guidelines, there is need for regular staff training, mentorship and motivation, and continuous monitoring of the implementation of guidelines. Enablers such as reliable sample and results transport systems, quality-assured testing laboratories and demand creation are essential to the success of routine VL testing scale up. In our study it was also revealed that paper-based results were sent to facilities using motorbikes. In this technological era, use of mHealth to provide same-day results through SMS technology will speed up the TAT and address the issue of unreliable transport to deliver results to facilities.

The delay in guideline dissemination results in delay in implementation of the policies with the result that some pregnant women living with HIV did not receive the preferred services. Being knowledgeable on a subject or policy does not necessarily translate to implementation of guidelines as there are other factors that contribute to successful implementation [36, 47].

Women who were interviewed indicated that they were provided with or received information during group education talks or during consultation. However, the women interviewed felt that the health education talks given while awaiting ANC were too brief and there was insufficient time for pregnant women to ask questions. In addition, though they appreciated the one-on-one consultation, they were still not given enough time to ask questions and discuss HIV-related issues including VL testing. Consultations were constrained by high patient volume. Van Bogaert et al*.* [48] reported similar findings whereby nurses indicated that information about diagnostics and treatment were brief, and patients’ questions and worries were neglected when the workload was heavy.

One of the best suggestions for improving VL testing practice that emerged from the nurse interviews was that VL sample collection be synchronised with either ANC visits or ARV resupply days. Some facilities provided outreach services in hard-to-reach areas, thereby improving access to care for some pregnant women living with HIV. Outreach services is one of the recognized differentiated service delivery models in provision of HIV services [49, 50].

### Strengths and limitations of this study

This descriptive cross-sectional study utilised collected facility-level data that reflects routine clinical practice [51]. Although the retrospective component used an adequately powered, well represented sample, the prospective interviews were conducted on small samples. Additionally, the interviews of PWLHIV were limited to 10 of the 15 research sites. Thus, the findings may not be truly representative of the health worker population and population of pregnant women living with HIV in Manicaland province.

Data deficits were also cited by the nurses and observed by the researchers during data abstraction and these data gaps from both paper-based registries and the electronic systems were attributed as a major contributor to the low VL testing coverages. Additionally, due to the retrospective design there were limitations in the availability and completeness of demographic and clinical data. Gaps in routine data has been well documented in studies that have utilised retrospective data sources [33, 36, 40], and these limitations impacted the quality of the data for such studies [19, 36, 40, 52, 53].

The cross-sectional design did not allow description of trends over time including the impact of the coronavirus disease 2019 pandemic on VL testing. Whether VL testing increased after 2018 as suggested by the response of 16 pregnant women living with HIV during the interviews could not be confirmed. Further study is needed to determine whether VL testing, and factors associated with VL testing have changed over time.

## Conclusions

The low rate of HIV VL testing among PWLHIV in Mutare district is cause for concern requiring urgent attention. The Ministry of Health should consider disseminating ARV guidelines/policy documents electronically which is quicker and may translate into faster implementation of new policies. There is need to conduct regular mentorship and establish quality improvement initiatives for PMTCT services. Alerts within the ePMS or on the files of PWLHIV may ensure that VL testing is done on time so that missed testing opportunities are minimised particularly in newly identified pregnant women living with HIV. Point of care technology and mHealth can reduce VL results TAT. Recommendations from the study can potentially contribute to improved provision of VL testing services among PWLHIV in Mutare district, the province and nationally.

## Supplementary Information

**Additional file 1: Table 1** Distribution of participants at the fifteen study sites in Mutare District, Zimbabwe

## Data Availability

The dataset used and/or analysed in this study is available from the corresponding author on reasonable request.

## Abbreviations

AIDS: Acquired Immunodeficiency syndrome
ANC: Antenatal care
ART: Antiretroviral therapy
ARV: Antiretroviral
aOR: Adjusted odds ratio
CI: Confidence interval
EDTA: Ethylenediamine tetra acetic acid
eMTCT: Elimination of mother to child transmission
ePMS: Electronic patient monitoring system
IQR: Interquartile range
HIV: Human Immunodeficiency Virus
mHealth: Mobile health
MSF: Médecins Sans Frontières
MTCT: Mother to child transmission
OR: Odds ratio
OPP-ERA: Open polyvalent platforms (OPP) for sustainable and quality access to VL in resource limited settings
PWLHIV: Pregnant women living with HIV
PCN: Primary care nurse
POC: Point of care
RGN: Registered general nurse
TAT: Turnaround time
UCT: University of cape town
UNICEF: United Nations Children’s Fund
UNAIDS: Joint United Nations Programme on HIV & AIDS
VL: Viral load
WHO: World Health Organisation
ZDHS: Zimbabwe demographic health survey
ZIMPHIA: Zimbabwe population-based HIV impact assessment
