# Supplementary material for: Viral load testing among pregnant women living with HIV in Mutare district of Manicaland province, Zimbabwe
Source: AIDS Res Ther. 2022 Nov 16;19:52. doi: 10.1186/s12981-022-00480-1 (PMC9667426; doi:10.1186/s12981-022-00480-1)
Supplement: Supplementary file 1 — Additional file 1: Table 1 Distribution of participants at the fifteen study sites in Mutare District, Zimbabwe [file 12981_2022_480_MOESM1_ESM.docx]

**Additional file**

**Additional file 1: Table 1** Distribution of participants at the fifteen study sites in Mutare District, Zimbabwe

| **Study site** | **Number of pregnant women living with HIV included in the retrospective analysis** | **Number of nurses interviewed** | **Number of pregnant women living with HIV interviewed** |
| --- | --- | --- | --- |
| Burma Valley Clinic (rural)* | 17 | 1 | 2 |
| Chikanga Clinic (urban)* | 27 | 1 | 0 |
| Chitakatira Clinic (rural)* | 45 | 1 | 0 |
| Chipfatsura Clinic (rural)* | 7 | 1 | 2 |
| Dangamvura Clinic (urban)* | 29 | 1 | 2 |
| Dora Clinic (rural)* | 31 | 0 | 0 |
| Marange Rural Hospital** | 35 | 1 | 3 |
| Mount Zuma Clinic* | 11 | 1 | 1 |
| Mutare Provincial Hospital (urban)*** | 0 | 0 | 0 |
| Nzvenga Clinic (rural)* | 13 | 0 | 0 |
| Rowa Clinic (rural)* | 11 | 1 | 2 |
| Sakubva Clinic (urban)* | 32 | 1 | 2 |
| St Werburghs Clinic (rural)* | 10 | 1 | 2 |
| St Joseph's Mission Hospital (rural)** | 32 | 1 | 2 |
| Zimunya Clinic (peri-urban)* | 83 | 1 | 1 |
| **Total** | **383** | **12** | **19** |

Level of service delivery: *clinic - level 1, **Rural or District level – level 2, ***Provincial hospital – level 3
